# Anaesthetic Management of Diabetic Ketoacidosis (DKA) in a Cesarean Section: A Case Report

**DOI:** 10.7759/cureus.51014

**Published:** 2023-12-23

**Authors:** Vishnu Priya, Sanjot Ninave, Jayshree Sen, Amol Bele

**Affiliations:** 1 Anaesthesiology, Jawaharlal Nehru Medical College, Datta Meghe Institute of Higher Education & Research, Wardha, IND

**Keywords:** maternal-fetal health, type 1 diabetes mellitus, multidisciplinary care, cesarean section, pregnancy, diabetic ketoacidosis

## Abstract

Diabetic ketoacidosis (DKA) is a life-threatening complication of diabetes mellitus that poses unique challenges during pregnancy. We present a case of a 36-year-old pregnant woman with a history of type 1 diabetes mellitus who developed severe DKA at 33.5 weeks of gestation, necessitating an emergency cesarean section. Despite a known history of diabetes, the patient's infrequent clinic attendance and suboptimal disease management contributed to her critical condition. DKA was promptly diagnosed, and a multidisciplinary team comprising obstetricians, endocrinologists, anesthesiologists, and neonatologists collaborated to provide comprehensive care. The preoperative assessment revealed dehydration and electrolyte imbalances, necessitating meticulous planning for IV fluid administration and hemodynamic stability during the cesarean section. Regional anaesthesia was chosen as the anaesthetic approach, and close postoperative monitoring was initiated. The neonate, delivered with satisfactory Apgar scores, was transferred to the neonatal ICU for observation. The patient's gradual clinical improvement over 48 hours demonstrated the importance of ongoing care. This case highlights the significance of early recognition, multidisciplinary teamwork, and meticulous perioperative care in managing DKA during pregnancy, ensuring favourable outcomes for both the mother and the neonate.

## Introduction

Diabetic ketoacidosis (DKA) is a severe and potentially life-threatening metabolic complication of diabetes mellitus. When DKA occurs during pregnancy, it presents a unique set of challenges, particularly when a cesarean section is required. Pregnant individuals with diabetes, especially those with preexisting type 1 diabetes, are at increased risk for DKA due to hormonal changes and increased insulin resistance during pregnancy [1]. This case report sheds light on the intricacies of managing DKA during late pregnancy, emphasising the importance of early recognition, multidisciplinary collaboration, and meticulous perioperative care.

The incidence of DKA during pregnancy is relatively low but can have devastating consequences for both the mother and the fetus [2]. Timely diagnosis and appropriate management are paramount to mitigate risks and optimise outcomes. This case report highlights the essential role of various medical specialists, including obstetricians, endocrinologists, anesthesiologists, and neonatologists, in coordinating care and addressing the complexities of DKA in a pregnant patient [3]. Through this case, we explore the challenges faced when managing DKA in the context of a cesarean section, the impact of DKA on maternal and neonatal health, and the strategies employed to ensure a successful outcome. Early recognition, comprehensive planning, and close postoperative monitoring are underscored to remind us of the significance of proactive and collaborative care in such critical medical situations.

## Case presentation

A 36-year-old female patient, who is Gravida 5, Para 1, with a history of three previous abortions and is currently at 33.5 weeks gestation, presented to the emergency department. She has a known history of type 1 diabetes mellitus, which has been present for seven years. The patient complained of nausea, vomiting, and shortness of breath over the past two days. Her blood glucose levels were markedly elevated at 500 mg/dL, and arterial blood gas analysis revealed the following results: a potential of hydrogen (pH) of 7.250, partial pressure of carbon dioxide (pCO^2^) of 35.3 mm Hg, base deficit of -14, and bicarbonate (HCO^3^) of 14.9. Additionally, urine ketones were measured at 3+, indicating metabolic acidosis consistent with diabetic ketoacidosis (DKA). All other laboratory investigations returned within normal limits. Notably, she had been previously admitted for diabetes-related issues one year ago. She had been an infrequent attendee at the diabetes clinic, which led to suboptimal management of her condition.

Upon examination, her vital signs included a respiratory rate of 26 breaths per minute and a pulse rate of 112 beats per minute. She displayed signs of dehydration, including sunken eyes and a dry mouth. Systemic examination revealed no other signs of infection. The patient was diagnosed with DKA, and she was promptly initiated on IV fluids, insulin therapy, and electrolyte replacement by established DKA management protocols. A bolus of 75 bicarbonate was administered, followed by 175 via IV drip over 12 hours. Subsequent investigations are shown in Table 1.

**Table 1:** The patient's laboratory investigation results

Parameter	Result	Normal range
Glycated hemoglobin (HbA1c) level	13	<5.7%
Blood glucose	350 mg/dL	70-99 mg/dL
Haemoglobin (Hb)	12.9	12-16 g/dL
Total leukocyte count	12,400	4,000-11,000/μL
Platelet count	1.56 l/L	150,000-450,000/μL
Sodium level	135 mmol/L	135-145 mmol/L
Potassium level	4.6 mmol/L	3.5-5.1 mmol/L
Blood urea	16	7-20 mg/dL
Creatinine	0.9	0.6-1.1 mg/dL
Activated partial thromboplastin time (APTT)	31	25-35 seconds
Prothrombin time and international normalized ratio (PT/INR)	1.01	0.9-1.1
Urine ketones	2+	Negative

A multidisciplinary team, which included an obstetrician, endocrinologist, anesthesiologist, and neonatologist, collaborated in the patient's care. The preoperative assessment revealed a dehydrated patient with tachycardia and hypotension. The anaesthetic plan was thoughtfully developed, involving the administration of adequate IV fluids to correct dehydration and maintain hemodynamic stability. Frequent monitoring of electrolyte levels was carried out to prevent further disturbances. Close surveillance of electrolytes and blood glucose levels aimed to prevent electrolyte imbalances and hypoglycemia during the perioperative period. Arterial blood gas analysis was performed to assess and manage the patient's acid-base status. Regional anaesthesia was chosen as the preferred anaesthetic approach. The neonatologist was present to evaluate and address the potential impacts of maternal DKA on the newborn.

**Figure 1:**
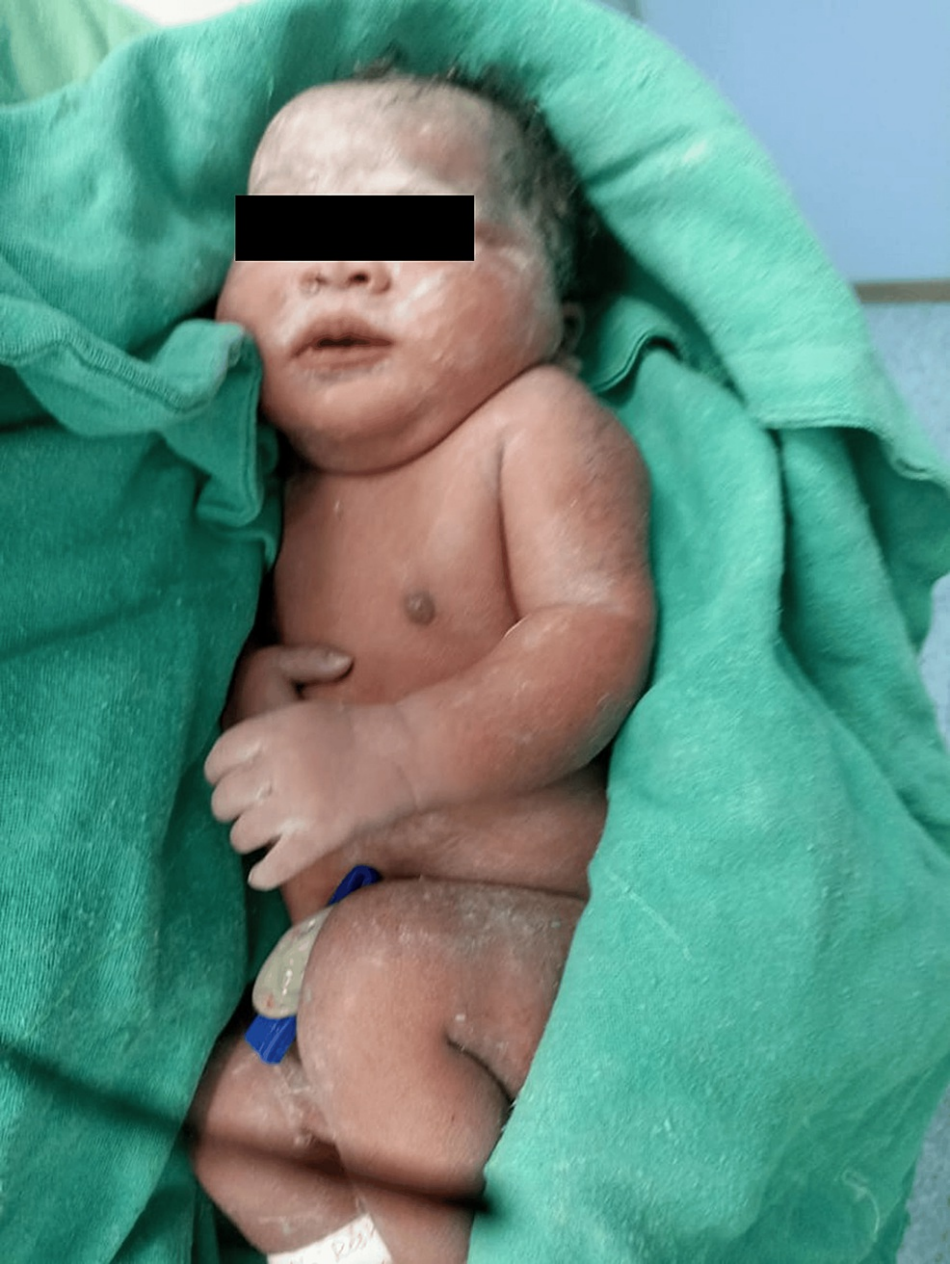
The successful outcome of a newborn after anaesthetic management

The cesarean section proceeded without complications under aseptic subarachnoid block anaesthesia. The infant was delivered with Apgar scores of 8 and 9 at one and five minutes, respectively (Figure 1). Minimal respiratory support was required, and the newborn was transferred to the neonatal ICU for observation. Postoperative analgesia was provided through a bilateral transverse abdominis block using 20 ml of 0.125% bupivacaine.

The patient's glucose levels were closely monitored in the postoperative period, and insulin therapy was adjusted as needed. The bicarbonate drip was continued to manage her acid-base balance. Over 48 hours, there was a gradual improvement in her clinical condition, and she was eventually discharged on the 11th day following delivery.

## Discussion

The presented case of a pregnant patient with DKA during the third trimester highlights the unique challenges and considerations in managing this complex medical condition in the context of a cesarean section. DKA during pregnancy is associated with increased maternal and fetal risks, making a multidisciplinary approach crucial [3]. This case emphasises the critical role of timely recognition and diagnosis. The patient's symptoms of nausea, vomiting, shortness of breath, and markedly elevated blood glucose levels indicated DKA. The arterial blood gas analysis revealed a severe metabolic acidosis consistent with DKA. It is imperative for healthcare providers to promptly recognise these clinical signs and laboratory findings, as delayed diagnosis and treatment can lead to severe complications [4].

Managing DKA during pregnancy is complex and requires a collaborative effort among various specialists. The involvement of an obstetrician, endocrinologist, anesthesiologist, and neonatologist ensured comprehensive care [5]. The preoperative assessment identified the patient's dehydration, tachycardia, and hypotension, guiding the anaesthetic plan. Adequate IV fluid administration was crucial in correcting dehydration and maintaining hemodynamic stability, as hyperglycemia-induced osmotic diuresis is a common contributor to dehydration in DKA [6]. Frequent monitoring of electrolytes and blood glucose levels was essential to prevent further derangements, as electrolyte imbalances can exacerbate the metabolic acidosis associated with DKA [7]. In this case, regional anaesthesia was a prudent choice, given the patient's condition and the potential risks of general anaesthesia in the context of DKA. Regional anaesthesia has been shown to have favourable outcomes in pregnant patients with DKA undergoing cesarean section [8].

The neonatologist's presence at delivery is significant in assessing and managing the newborn, as maternal DKA can impact the neonate. The Apgar scores at one and five minutes were reassuring, suggesting that the neonate did not experience severe distress. However, careful observation in the neonatal ICU was still warranted, given the potential risks associated with maternal DKA [9]. In the postoperative period, the close monitoring of glucose levels and the need for insulin therapy adjustments highlight the importance of ongoing care and vigilance. The patient's gradual clinical improvement over 48 hours indicates that with proper management, patients can recover successfully from DKA during pregnancy [10].

## Conclusions

This case report underscores the challenges and complexities of managing DKA in pregnant patients, particularly when requiring a cesarean section. The presented patient, with a history of suboptimal diabetes management, presented with severe DKA at 33.5 weeks gestation. A multidisciplinary approach involving an obstetrician, endocrinologist, anesthesiologist, and neonatologist was pivotal in ensuring a successful outcome. The preoperative assessment identified critical factors, such as dehydration and electrolyte imbalances, necessitating meticulous planning to maintain hemodynamic stability. The choice of regional anaesthesia proved suitable, considering the patient's condition. The diligent monitoring of glucose levels and electrolyte status in the postoperative period, alongside prompt insulin therapy adjustments, was vital. The gradual clinical improvement observed over 48 hours highlights the importance of comprehensive, collaborative care in managing complex cases of DKA during pregnancy. This case serves as a reminder of the significance of early recognition, prompt intervention, and multidisciplinary teamwork in optimising outcomes for pregnant patients with DKA.

## References

[1] Syed M, Javed H, Yakoob MY, Bhutta ZA (2011). Effect of screening and management of diabetes during pregnancy on stillbirths. BMC Public Health.

[2] Sibai BM, Viteri OA (2014). Diabetic ketoacidosis in pregnancy. Obstet Gynecol.

[3] Kamalakannan D, Baskar V, Barton DM, Abdu TA (2003). Diabetic ketoacidosis in pregnancy. Postgrad Med J.

[4] Kitabchi AE, Umpierrez GE, Miles JM, Fisher JN (2009). Hyperglycemic crises in adult patients with diabetes. Diabetes Care.

[5] Dargel S, Schleußner E, Kloos C, Groten T, Weschenfelder F (2021). Awareness of euglycaemic diabetic ketoacidosis during pregnancy prevents recurrence of devastating outcomes: a case report of two pregnancies in one patient. BMC Pregnancy Childbirth.

[6] Gosmanov AR, Gosmanova EO, Kitabchi AE (2000). Hyperglycemic crises: diabetic ketoacidosis and hyperglycemic hyperosmolar state. Endotext [Internet].

[7] Buhary BM, Almohareb O, Aljohani N (2016). Glycemic control and pregnancy outcomes in patients with diabetes in pregnancy: a retrospective study. Indian J Endocrinol Metab.

[8] Bryant SN, Herrera CL, Nelson DB, Cunningham FG (2017). Diabetic ketoacidosis complicating pregnancy. J Neonatal Perinatal Med.

[9] Mathiesen ER, Ringholm L, Damm P (2011). Stillbirth in diabetic pregnancies. Best Pract Res Clin Obstet Gynaecol.

[10] Serpa Neto A, Nassar AP, Cardoso SO (2012). Vasopressin and terlipressin in adult vasodilatory shock: a systematic review and meta-analysis of nine randomized controlled trials. Crit Care.

